# ^**18**^ **F-ASEM PET/MRI targeting alpha7-nicotinic acetylcholine receptor can reveal skeletal muscle denervation**

**DOI:** 10.1186/s13550-024-01067-9

**Published:** 2024-01-22

**Authors:** Yong-il Kim, Seung Hak Lee, Jin Hwa Jung, Seog-Young Kim, Nare Ko, Sang Ju Lee, Seung Jun Oh, Jin-Sook Ryu, Dabin Ko, Won Kim, Kyunggon Kim

**Affiliations:** 1 Department of Nuclear Medicine, Asan Medical Center, University of Ulsan College of Medicine, Seoul, Republic of Korea; 2 Department of Rehabilitation Medicine, Asan Medical Center, University of Ulsan College of Medicine, 88 Olympic-Ro 43-Gil, Songpa-Gu, Seoul, 05505 Republic of Korea; 3 Convergence Medicine Research Center, Asan Institute for Life Sciences, Asan Medical Center, Seoul, Republic of Korea; 4 Asan Institute for Life Sciences, Asan Medical Center, Seoul, Republic of Korea; 5 Department of Biomedical Sciences, University of Ulsan College of Medicine, Seoul, Republic of Korea; 6 Clinical Proteomics Core Laboratory, Convergence Medicine Research Center, Asan Medical Center, Seoul, Republic of Korea

**Keywords:** Muscle denervation, Peripheral nervous system, Nicotinic acetylcholine receptor, ^18^F-ASEM, Positron emission tomography

## Abstract

**Background:**

The increased expression of the nicotinic acetylcholine receptor (nAChR) in muscle denervation is thought to be associated with electrophysiological acetylcholine supersensitivity after nerve injury. Hence, we investigated the utility of the ^18^F-ASEM alpha7-nAChR targeting radiotracer as a new diagnostic method by visualizing skeletal muscle denervation in mouse models of sciatic nerve injury.

**Methods:**

Ten-week-old C57BL/6 male mice were utilized. The mice were anesthetized, and the left sciatic nerve was resected after splitting the gluteal muscle. One week (n = 11) and three weeks (n = 6) after the denervation, ^18^F-ASEM positron emission tomography/magnetic resonance imaging (PET/MRI) was acquired. Maximum standardized uptake values (SUVmax) of the tibialis anterior muscle were measured for the denervated side and the control side. Autoradiographic evaluation was performed to measure the mean counts of the denervated and control tibialis anterior muscles at one week. In addition, immunohistochemistry was used to identify alpha7-nAChR-positive areas in denervated and control tibialis anterior muscles at one week (n = 6). Furthermore, a blocking study was conducted with methyllycaconitine (MLA, n = 5).

**Results:**

^18^F-ASEM PET/MRI showed significantly increased ^18^F-ASEM uptake in the denervated tibialis anterior muscle relative to the control side one week and three weeks post-denervation. SUVmax of the denervated muscles at one week and three weeks showed significantly higher uptake than the control (*P* = 0.0033 and 0.0277, respectively). The relative uptake by autoradiography for the denervated muscle was significantly higher than in the control, and immunohistochemistry revealed significantly greater alpha7-nAChR expression in the denervated muscle (*P* = 0.0277). In addition, the blocking study showed no significant ^18^F-ASEM uptake in the denervated side when compared to the control (*P* = 0.0796).

**Conclusions:**

Our results suggest that nAChR imaging with ^18^F-ASEM has potential as a noninvasive diagnostic method for peripheral nervous system disorders.

**Supplementary Information:**

The online version contains supplementary material available at 10.1186/s13550-024-01067-9.

## Background

A variety of diseases can affect the peripheral nervous system. Peripheral nerve trauma [1] and neuromuscular diseases [2] (e.g., amyotrophic lateral sclerosis) directly cause damage to the peripheral nervous system, and systemic diseases, such as diabetes [3]*,* can also cause peripheral nervous system abnormalities. Patients with these diseases have neurological deficits, including motor weakness and sensory deficits, which lead to a significant deterioration in their quality of life [4]. The diagnosis and evaluation of these diseases are clinically very important, and electrophysiological studies, including needle electromyography (nEMG), are the most important evaluation tools [2, 5]. nEMG can be used to detect unstable myocyte membrane potential, which indicates muscle denervation after peripheral neuromuscular compromise [6]. However, nEMG has several critical weaknesses. Multiple needle insertions and explorations in muscle tissue are necessary to accomplish the examination; as a result, patients will inevitably experience serious pain [7]. In addition, the interpretation of nEMG is significantly subjective, and the opinion of an experienced electromyographer is crucial. Even though several imaging modalities, such as magnetic resonance imaging (MRI) [8, 9] and ^18^F-fluorodeoxyglucose (FDG) positron emission tomography (PET) [10–12], can also be used to evaluate these diseases, nEMG is still the current gold-standard method.

After denervation, the skeletal muscle undergoes morphological, physiological, and molecular changes. The development of acetylcholine supersensitivity is one of these changes, and the overexpression of nicotinic acetylcholine receptor (nAChR) outside the end-plate region after denervation is suggested as an underpinning molecular mechanism [13]. This phenomenon is accompanied by the increased expression of several nAChR subtypes, such as embryonic and neuronal types [14]. Alpha7-nAChR is a neuronal type that also exhibits increased expression in skeletal muscle after denervation [15, 16]. Some radiotracers that target nAChR (such as ^11^C-nicotine and ^18^F-A85380) have been developed; however, they demonstrated high non-specific binding [17]. Recently, great progress has been made in developing alpha7-nAChR targeting radiotracers, including ^11^C-CHIBA-1001, ^18^F-DBT-10, and ^18^F-ASEM [18]. Among them, ^18^F-ASEM is currently widely used due to its high specificity and sufficient binding activity [19]. Therefore, we hypothesized that ^18^F-ASEM PET/MRI could provide electrophysiological information through visualization of the denervated skeletal muscle. This novel imaging technique could be used to diagnose and evaluate diseases of the peripheral nervous system.

The purpose of this study was to confirm the visualization of skeletal muscle denervation using alpha7-nAChR PET/MRI in a sciatic nerve injury mouse model and to investigate the feasibility of this imaging technique as a noninvasive imaging diagnostic tool for peripheral nervous system disorders.

## Methods

### Denervation model

A total of 30 C57BL/6 mice (10-weeks-old; male) were used for this study (Additional file 1: Fig. S1). Complete sciatic nerve transections were performed to establish skeletal muscle denervation. The mice were anesthetized with 2% isoflurane inhalation and the gluteal muscles were dissected to expose the left sciatic nerve. A 5 mm long nerve segment was resected in the proximal part of the nerve before it bifurcated into the common peroneal and tibial nerves. The experimental protocol was approved by the Institutional Animal Care and Use Committee (IACUC) of the Asan Medical Center (IACUC approval no. 2020–12-081).

### Radiosynthesis of ^18^F-ASEM

The ASEM precursor (3-(1,4-diazabicyclo [3.2.2] nonan-4-yl)-6-nitrodibenzo [b, d] thiophene 5,5-dioxide) and the radiotracer ^18^F-ASEM were prepared as described in a previously reported study with minor modifications [20]. Briefly, ^18^F-fluoride (F^−^) (85.7 ± 14.3 GBq) produced by a GE PETtrace cyclotron was trapped on a Sep-Pak Accell Plus QMA Plus Light Cartridge (Waters Corporation, Milford, MA, USA) and eluted using a solution of Kryptofix 222 (22 mg), 0.2 M potassium methanesulfonate (0.1 mL), and methanol (1 mL) [21]. The eluted ^18^F-F^−^ solution was transferred to a reaction vial of Trasis All-In-One synthesizer (Ans, Belgium) and heated at 100 °C under an N_2_(g) stream. For azeotrope evaporation, 1 mL of acetonitrile was added to the reactor during the heating process. After drying, a solution of ASEM precursor (1 mg) dissolved in anhydrous dimethyl sulfoxide (1 mL) was added to the reactor and heated at 150 °C for 10 min. The reaction mixture was purified and reformulated with high-performance liquid chromatography and a Sep-Pak C18 Plus Short Cartridge (Waters Corporation, Milford, MA, USA). The average radiochemical purity and the specific radioactivity of the ^18^F-ASEM were determined to be > 99% and 260.3 ± 67.6 GBq/μmol (7.0 ± 1.8 Ci/μmol) calculated at the end of the synthesis, respectively (n = 3). The average radiochemical yield was 6.2 ± 0.6% (non-decay corrected), and the total preparation time was 60–70 min.

### ^18^F-ASEM PET/MRI and Analysis

The mice underwent small animal PET/MRI using a nanoScanPET/MRI system (1 T, Mediso, Hungary) 1 week (n = 13; 2 for the dynamic study and 11 for the static study) and 3 weeks (n = 6) after the denervation. Under anaesthesia (1.5% isoflurane in 100% O_2_ gas), whole-body MRI was used to obtain T1 weighted with gradient-echo three-dimensional (3D) sequence (TR = 25 ms, TE_eff_ = 3.4, FOV = 64 mm, matrix = 220 × 220) images approximately 12 min before the injection of ^18^F-ASEM. After completion of the MRI, 7.3 ± 1.4 MBq of ^18^F-ASEM (dissolved in 0.2 mL of normal saline) was administered intravenously via the tail vein and 60 min of dynamic PET and 20 min of static PET images were acquired in a 1–5 coincidence range in a single field of view with the MRI. Body temperature was maintained by heating the air on the animal bed (Multicell, Mediso, Hungary), and a pressure-sensitive pad was used for respiratory triggering. PET images were reconstructed by Tera-Tomo 3D, in full detector mode, with all of the corrections on, high regularization, and eight iterations. A 3D volume of interest (VOI) analysis of the reconstructed images was performed using the InterView Fusion software package (Mediso, Hungary) and applying standardized uptake value (SUV) analysis. VOI, fixed with a diameter of a 2 mm sphere, was first drawn for the left tibialis anterior muscle to identify the highest SUV lesion in the muscle. After that, another VOI was drawn for the corresponding contralateral right tibialis anterior muscle. The SUV of each VOI site was calculated using the formula: SUV = (tumor radioactivity in the tumor VOI with the unit of Bq/mL × body weight [g]) divided by the injected radioactivity.

### Autoradiography

After ^18^F-ASEM PET/MRI for the 1-week model, the animals were processed for autoradiography. Target tissues were rapidly isolated, submerged in Tissue-Tek O.C.T. compound with Tissue-Tek cryomold (Sakura Finetek USA, Inc., CA, USA), and frozen slowly in isopentane at − 20 to − 30 °C. Serial 10 μm tissue sections were cut with a cryostat (CM3050S, Leica Biosystems, Wetzlar, Germany) and placed in a cassette under phosphor imaging plates (BAS-SR, Cytiva, MA, USA) for an exposure period of 24 h, which were read on an Amersham Typhoon plate reader (Cytiva, USA) at a 25 μm pixel resolution. Quantitative analysis was performed using ImageQuant TL Toolbox (v8.2.0, Cytiva, USA) with the circle region of interest (ROI) at the centre region, covering all conceivable tissue areas. To determine the relative uptake levels, quantitative data from the denervated group were normalized to the corresponding values of the control group.

### nEMG and immunohistochemistry

A total of six denervation 1-week mice were anesthetized with 2% isoflurane for nEMG. A two-channel portable electrodiagnostic system (UltraPro S100, Natus Neurology Inc., RI, USA) was used for the nEMG. A monopolar electrode (Disposable Monopolar Needles, Natus Neurology, USA) was inserted into the lower leg muscles to confirm membrane instability after anesthetization. At four points in the lower leg muscles, fibrillation and positive sharp waves were checked. After nEMG, the mice were sacrificed by cervical dislocation and the tibialis anterior muscles of both sides were harvested and placed in 4% paraformaldehyde overnight for fixation. The fixed tissues were rinsed with tap water for approximately 2 h to remove the formalin. The tissues were then dehydrated in graded ethanol, cleared in xylene using a tissue processor (Excelsior ES, Thermo C Shandon, UK), and embedded in paraffin blocks using a paraffin embedding station (EG1150H, Leica Biosystems, USA). Paraffin-embedded tissues were sectioned into 3 µm thick segments on a rotary microtome (RM2255, Leica Biosystems, USA) and mounted onto CREST glass slides (Matsunami, Japan). The immunohistochemistry staining was performed using a Ventana BenchMark XT immunostainer and UltraView Universal DAB Detection Kit (Ventana Medical Systems, AZ, USA). In brief, the tissue sections were warmed to 60 °C and incubated for 4 min. Slides were dewaxed in xylene and rehydrated through graded alcohols. Sections were rinsed twice with Tris buffer (pH 7.6) and incubated with Tris/borate/ EDTA buffer (pH 8.4) for antigen retrieval for 60 min at 100 °C. After rinsing the slides for 10 min in Tris buffer three times, endogenous peroxidase activity was blocked by incubating with 0.3% H_2_O_2_ in methanol for 4 min. The sections were blocked off and covered with the purified anti-alpha7-nAChR rabbit polyclonal primary antibody (#ANC-007, Alomone Labs, Israel), which was diluted at 1:100 in antibody diluent solution (GBI Labs, WA, USA) for 36 min at room temperature. Sections were washed with Tris buffer and incubated with the UltraMap anti-rabbit HRP conjugated secondary antibody (#760-4315, Ventana, USA) for 16 min at 37 °C. The incubated section was washed with Tris buffer, and Ultraview DAB and DAB H_2_O_2_ solution was used for 8 min at room temperature for the development of the brown colour. The slides were washed with Tris buffer and Ultraview Copper solution was applied for 4 min at room temperature. The specimens were counterstained with hematoxylin. A bluing reagent was used for post-counterstaining for 4 min at room temperature. Immunohistochemistry sections were digitally captured for quantitative analysis. Ten random locations were selected within the entire muscle area at a 200 × magnification. The quantification of alpha7-nAChR expression was performed using Image J software (NIH, MD, USA). The digital images were converted to grayscale and the percentage of the area stained with DAB was calculated. The threshold for staining positivity was uniformly set at 174 across all images.

### Blocking study

In the blocking group (n = 5), methyllycaconitine (MLA; Abcam, ab120072), an antagonist of alpha7-nAChR, was used [22]. MLA was diluted in 0.9% saline and intraperitoneally administered to the mice at a concentration of 10 mg/kg/day [23]. The volume of a single daily intraperitoneal injection was 100 µL. The surgery was performed on the 3rd day and ^18^F-ASEM PET/MRI was performed on the 10th day after the first administration of the blocking agent. The last administration was performed 10 min before imaging. The mice were sacrificed after imaging by cervical dislocation and both tibialis anterior muscles were harvested for autoradiography.

### Statistical analysis

Statistical analysis was performed using the Wilcoxon signed-rank test, with MedCalc Version 19.1.7 for Windows (Mariakerke, Belgium). A P-value less than 0.05 was considered statistically significant. We compared the relative uptake of autoradiography, and the relative uptake with 95% confidence intervals not including the value 1.0 were statistically significant (which means a P-value less than 0.05) [24]. Additionally, we drew Box-and-Whisker plots for visual comparisons of the study results. Boxes were drawn from the 1st and 3rd quartile values and a horizontal line was drawn at the median value. The highest/lowest value just below the upper/lower inner line was the upper/lower adjacent value and a horizontal line was drawn at this value. Vertical lines were drawn from the 3rd quartile to the upper adjacent value and the 1st quartile to the lower adjacent value. The small points represent the actual values.

## RESULTS

### ^18^F-ASEM PET/MRI of Denervation at one week

**Fig. 1:**
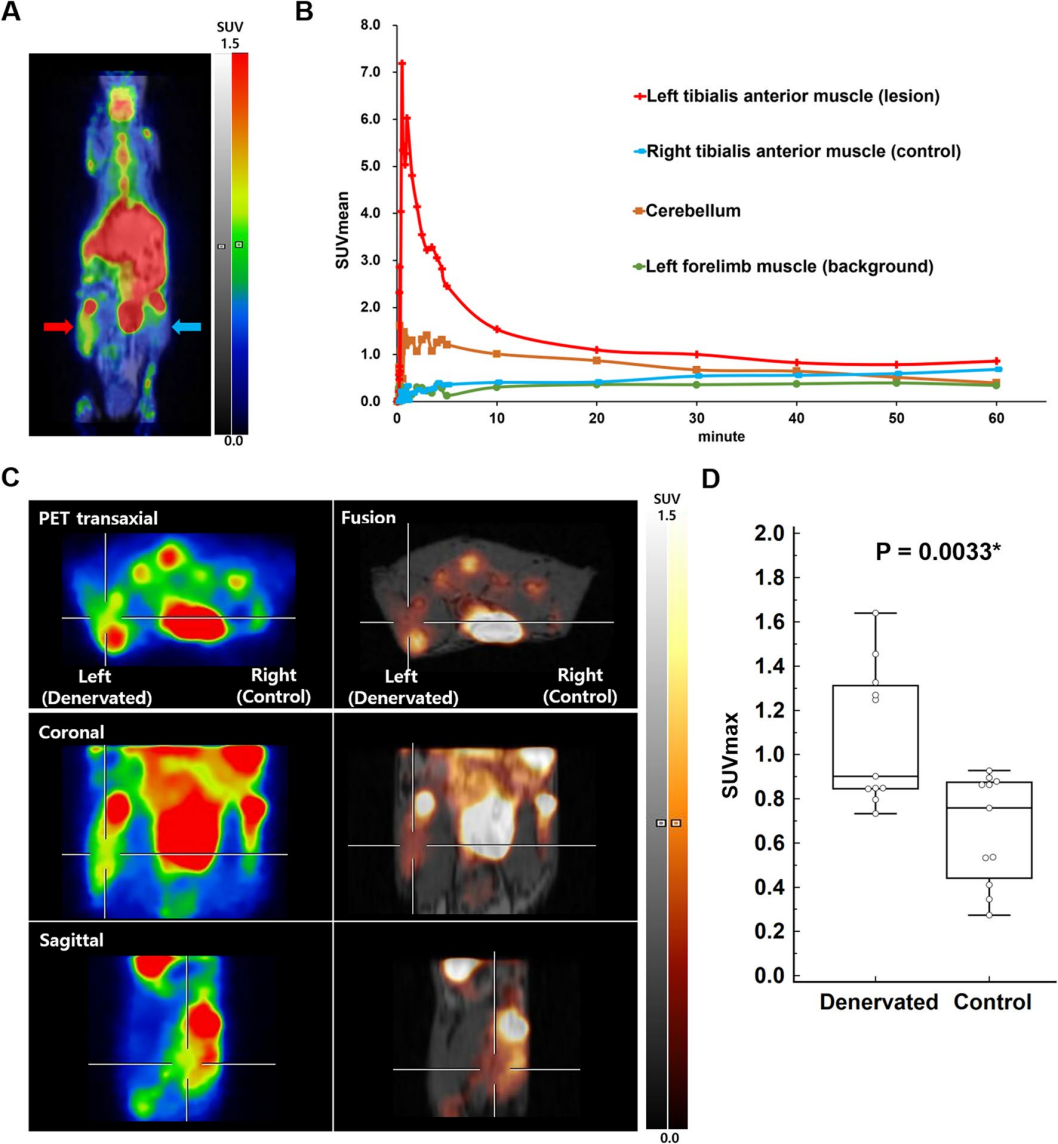
Denervation 1 week results of dynamic and static ^18^F-ASEM PET/MRI. **A** Dynamic ^18^F-ASEM PET/MRI showed increased uptake in the denervated left tibialis anterior muscle (red arrow) than in the control muscle (blue arrow). **B** Dynamic ^18^F-ASEM uptake by the denervated muscle slowly decreased after radiotracer injection. **C** Static ^18^F-ASEM PET/MRI also showed increased uptake in the denervated muscle (cross) than in the control muscle. The denervated muscle SUVmax was 1.33 and the control muscle SUVmax was 0.88. **D** The denervated muscle SUVmax of^18^F-ASEM PET/MRI was significantly higher than that of the control muscle (1.08 ± 0.31 vs. 0.66 ± 0.25, n = 11, *P* = 0.0033*)

A dynamic study of two mice showed increased ^18^F-ASEM uptake in the denervated left tibialis anterior muscle relative to the control muscle (Fig. 1A). As ^18^F-ASEM uptake in the denervated left tibialis anterior muscle slowly decreased after radiotracer injection, we set the static PET imaging time at 15–35 min after radiotracer injection (Fig. 1B). Static ^18^F-ASEM PET/MRI also showed increased uptake in the denervated left tibialis anterior muscle relative to the control muscle of the denervation model at one week (Fig. 1C). The lesion maximum SUV (SUVmax) and mean SUV (SUVmean) of ^18^F-ASEM PET/MRI were significantly higher than the control SUVmax and SUVmean (1.08 ± 0.31 vs. 0.66 ± 0.25, *P* = 0.0033; 0.89 ± 0.23 vs. 0.54 ± 0.20, *P* = 0.0033; n = 11, respectively) (Fig. 1D).

### Autography, nEMG, and immunohistochemistry

**Fig. 2:**
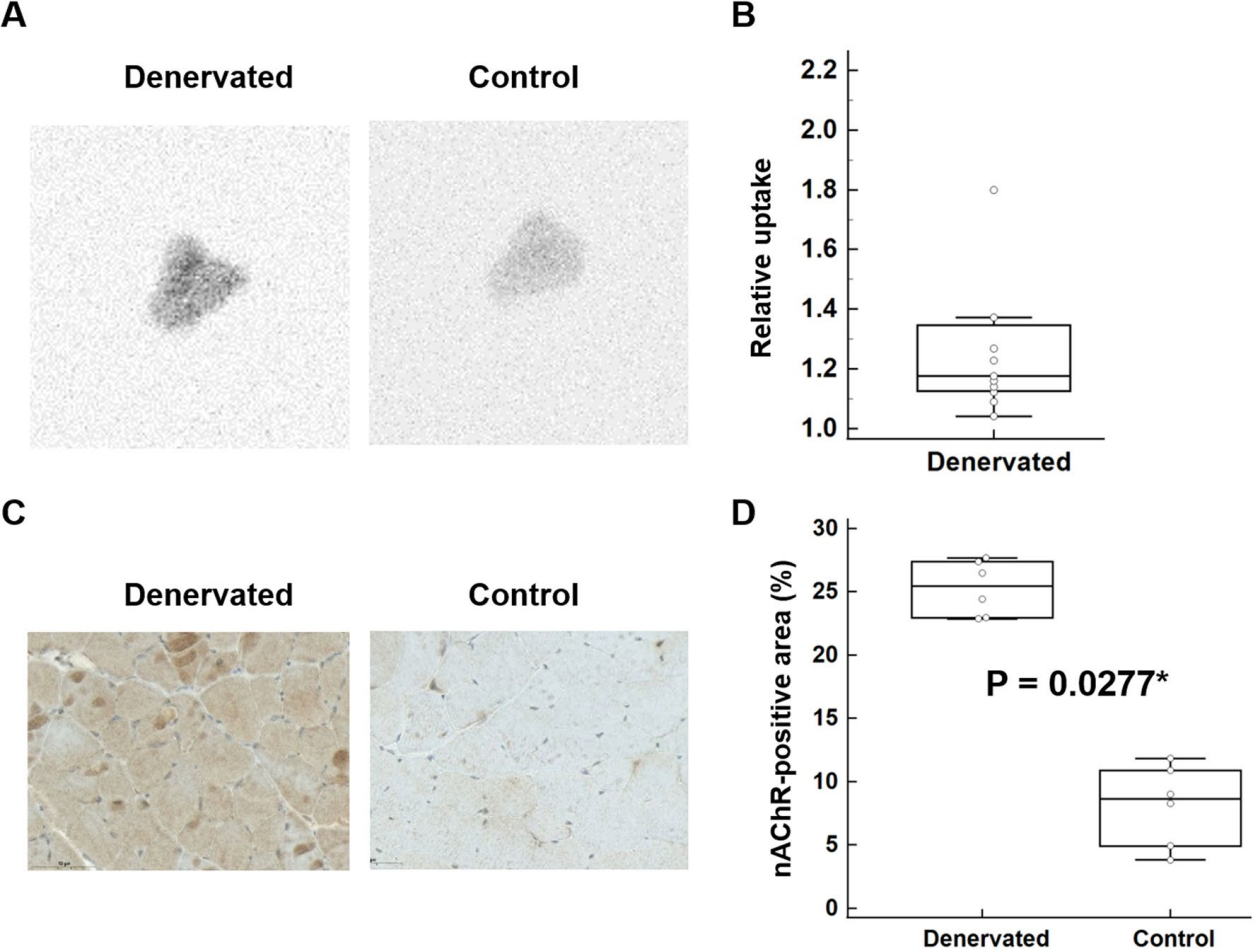
Autoradiography and immunohistochemistry results. **A**, **B** Autoradiography demonstrated significantly increased uptake in the denervated muscle than in the control (95% confidence interval 1.07–1.62, n = 11). **C**, **D** Immunohistochemistry revealed a significantly greater alpha7-nAChR-positive area in the denervated muscle than in the control (25.3 ± 2.17% vs. 8.13 ± 3.19%, n = 6, *P* = 0.0277*)

Autoradiography demonstrated a significantly increased uptake in the denervated tibialis anterior relative to the control (95% confidence interval 1.07–1.62, n = 11) (Fig. 2A, B). In addition, six 1 week denervation model mice showed abundant membrane instability in the left lower leg muscles by nEMG, which indicated skeletal muscle denervation after left sciatic nerve transection. Immunohistochemistry revealed a significantly larger alpha7-nAChR positive area in the denervated tibialis anterior muscle than in the control (25.3 ± 2.17% vs. 8.13 ± 3.19%, n = 6, *P* = 0.0277) (Figs. 2C, D).

### Blocking study

**Fig. 3:**
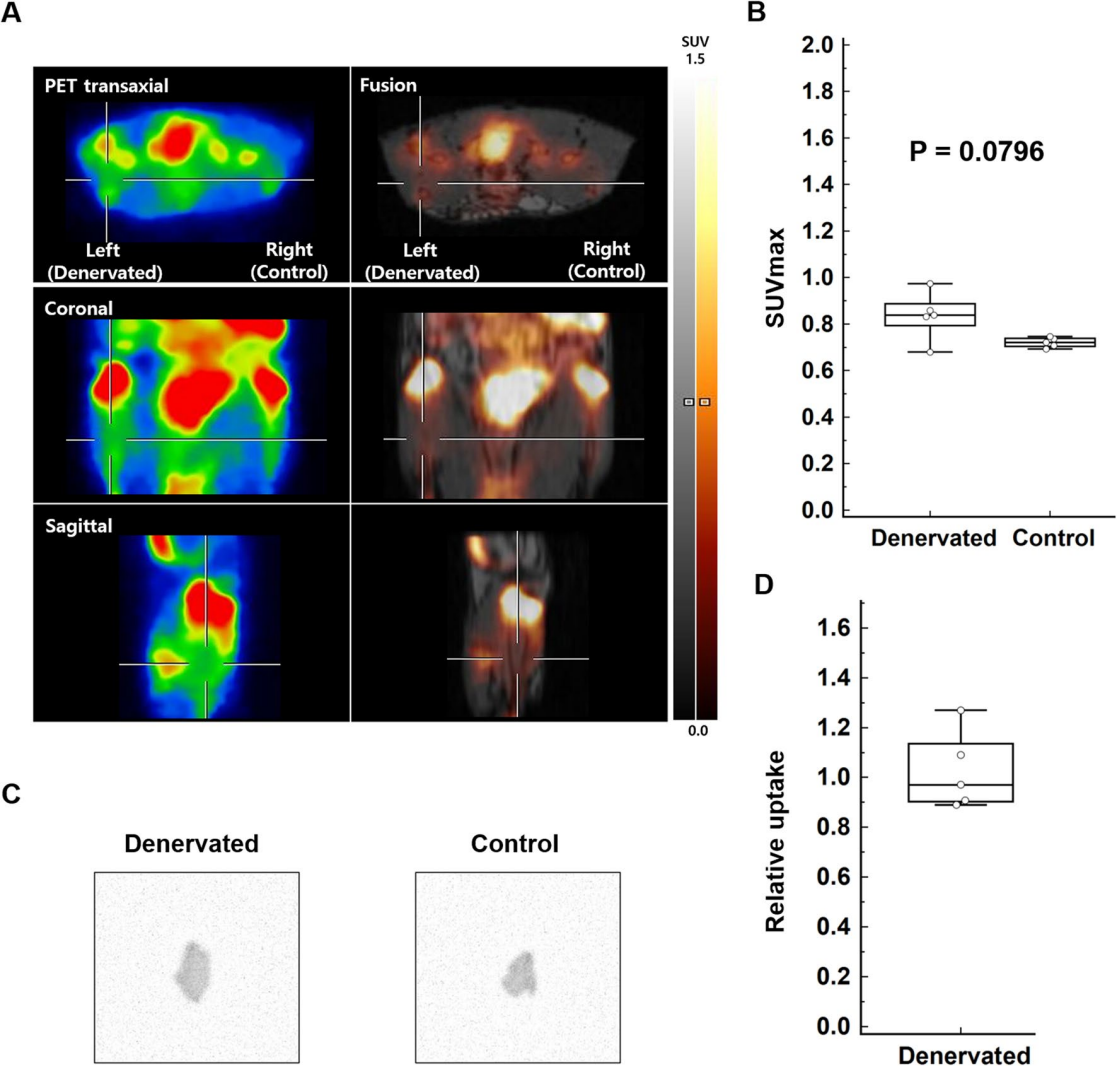
Blocking study results of ^18^F-ASEM PET/MRI and autoradiography. **A**, **B** ^18^F-ASEM PET/MRI showed no abnormally increased uptake in the denervated left tibialis anterior muscle (cross) relative to the control. The denervated muscle SUVmax was 0.68 and the control muscle SUVmax was 0.69. The denervated muscle SUVmax of.^18^F-ASEM PET/MRI was not significantly different from the control SUVmax (0.84 ± 0.10 vs. 0.72 ± 0.02, n = 5, *P* = 0.0796). **C**, **D** Autoradiography also demonstrated no significantly increased uptake in the denervated muscle relative to the control (95% confidence interval 0.83–1.22, n = 5)

We next investigated whether an inhibitor could antagonize the increased ^18^F-ASEM uptake after skeletal muscle denervation. After blocking with MLA, ^18^F-ASEM uptake in the denervated left tibialis anterior muscle was not evident compared to the control side (Fig. 3A). The lesion SUVmax and SUVmean of ^18^F-ASEM PET/MRI were not significantly different from the control SUVmax and SUVmean (0.84 ± 0.10 vs. 0.72 ± 0.02, *P* = 0.0796; 0.68 ± 0.10 vs. 0.62 ± 0.03, *P* = 0.1380; n = 5, respectively) (Fig. 3B). Autoradiography also demonstrated no significantly increased uptake in the denervated tibialis anterior relative to the control (95% confidence interval 0.83–1.22, n = 5) (Fig. 3C, D).

### ^18^F-ASEM PET/MRI at 3 weeks denervation

**Fig. 4:**
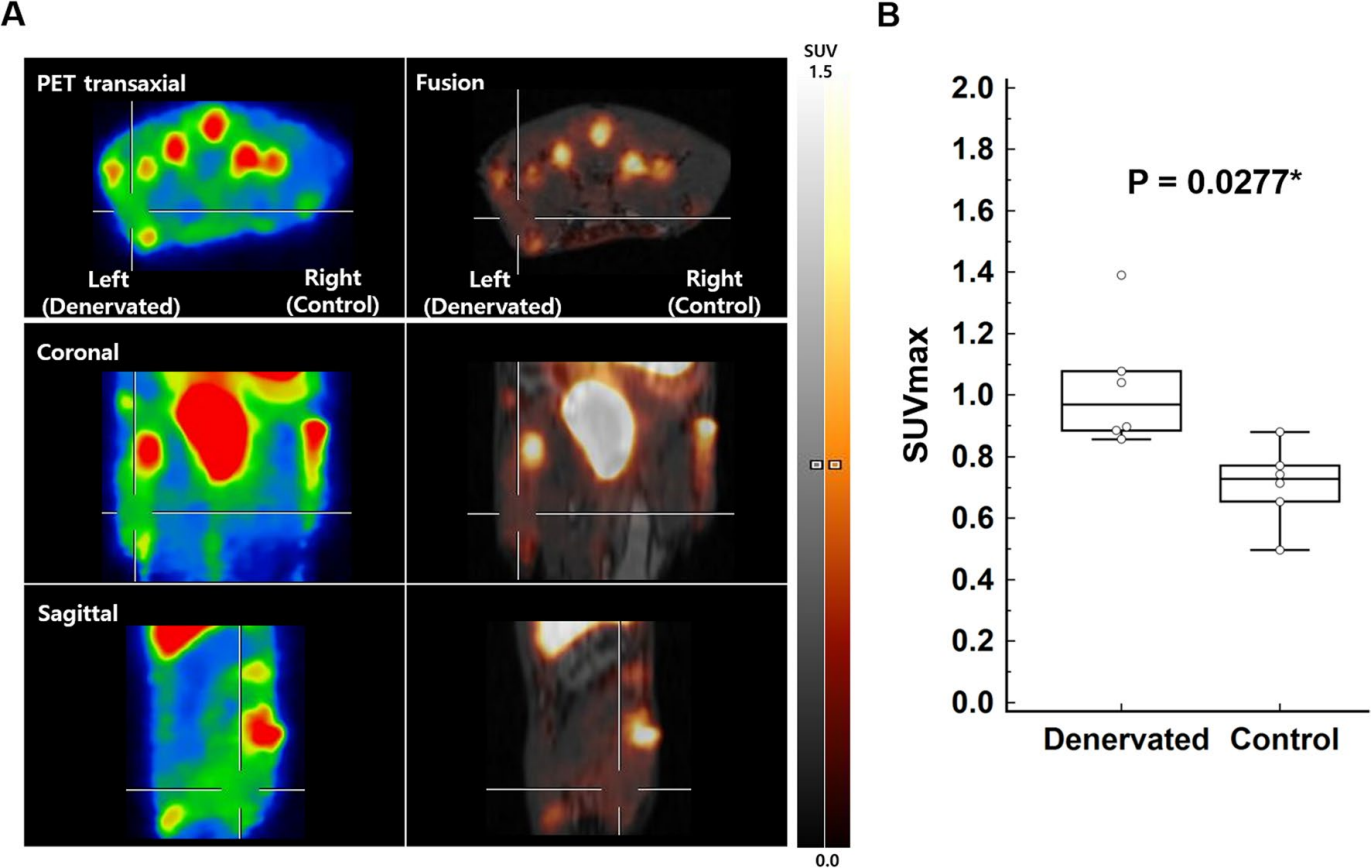
Denervation 3 week results of ^18^F-ASEM PET/MRI. **A** ^18^F-ASEM PET/MRI showed increased uptake in the denervated left tibialis anterior muscle (cross) relative to the control. The denervated muscle SUVmax was 1.39 and the control muscle SUVmax was 0.71. **B** The denervated muscle SUVmax of.^18^F-ASEM PET/MRI was significantly higher than that of the control muscle (1.02 ± 0.20 vs. 0.56 ± 0.12, n = 6, *P* = 0.0277*)

We established a denervation model using left sciatic transection. Three weeks later, ^18^F-ASEM PET/MRI showed increased uptake in the denervated left tibialis anterior muscle relative to the control muscle (Fig. 4A). The lesion SUVmax and SUVmean of ^18^F-ASEM PET/MRI were significantly higher than those of the control (1.02 ± 0.20 vs. 0.56 ± 0.12, *P* = 0.0277; 0.79 ± 0.18 vs. 0.56 ± 0.12, *P* = 0.0277; n = 6, respectively) (Fig. 4B). There was no obvious difference in the ^18^F-ASEM PET/MRI results between the denervation mouse models at 1 week and 3 weeks.

## Discussion

In this study, we found that: (i) alpha7-nAChR expression increased after denervation of the skeletal muscles, and (ii) alpha7-nAChR imaging via ^18^F-ASEM PET/MRI can visualize denervated skeletal muscles in a mouse sciatic transection model. If these results can be applied clinically, it will be possible to conduct objective and quantitative evaluations, avoiding painful nEMG tests, for patients with various peripheral nervous system disorders. To the best of our knowledge, this is the first study to investigate the use of ^18^F-ASEM in diagnosing peripheral nervous system disorders.

We first tried dynamic ^18^F-ASEM PET/MRI to identify the biodistribution of the tracer in muscles. After radiotracer injection with an initial variation, the uptake in the denervated left tibialis anterior muscle slowly decreased and the uptake in the control muscle slowly increased, and consequently, we set our static ^18^F-ASEM PET/MRI as occurring between 15 and 35 min. Compared with previous studies of the ^18^F-ASEM biodistribution in the human brain, the excretion was rather fast [25, 26]. However, a recent whole-body biodistribution study of ^18^F-ASEM showed that muscle is the lowest uptake site of the body and it could show different kinetics than the highest uptake site of the body (the brain) [27].

The significant static ^18^F-ASEM PET/MRI findings support our hypothesis that it can be used to visualize denervated skeletal muscle. We also identified increased ^18^F-ASEM uptake by autoradiography and increased nAChR expression by immunohistochemistry in skeletal muscle (with the denervation proven by nEMG). Furthermore, the blocking study demonstrated that ^18^F-ASEM uptake was real uptake by alpha7-nAChR rather than inflammatory or non-specific uptake. Finally, the increased ^18^F-ASEM uptake in the skeletal muscles persisted at 3 weeks, indicating that the increase in alpha7-nAChR expression is maintained after denervation. In this experiment, we utilized a complete nerve injury model, and based on previous studies of this model, we expected no recovery over the 3-week period. Skeletal muscle denervation involves a dynamic process of fatty atrophy, suggesting a sustained increase in the expression of alpha7-nAChR. Previous studies in rodent models using ^18^F-fludeoxyglucose have observed glucose hypermetabolism in denervated skeletal muscle for up to 12 weeks [10].

Until now, ^18^F-ASEM research was mainly limited to the brain. ^18^F-ASEM uptake in the brain was increased in patients with mild cognitive disorders [28] and ^18^F-ASEM uptake in the hippocampus was decreased in patients with psychosis [29]. In addition, ^18^F-ASEM uptake in the human cingulate cortex, frontal cortex, and hippocampus was decreased in patients with schizophrenia [30]. In recent years, ^18^F-ASEM has been investigated in other disorders unrelated to the brain. For example, alpha7-nAChR is an important target for identifying vulnerable atherosclerotic plaques [31] and was visualized in the carotid arteries of mice [32]. We expect that our study results could expand the investigations of this alpha7-nAChR receptor targeting radiotracer to many fields beyond brain disorders.

Our study had several limitations. First, sciatic transection is an extreme traumatic nerve injury model and cannot represent peripheral nerve injuries of different types and severities. Translational research following the temporal course of alpha7-nAChR expression in muscle denervation according to nerve injury severity and uptake intensity changes of ^18^F-ASEM is required. Second, increased alpha7-nAChR expression in skeletal muscles could be an unspecific phenomenon of denervation. Direct skeletal muscle injury and wound healing processes upregulated alpha7-nAChR expression in a rat model [33]. This issue is related to the sensitivity and specificity of alpha7-nAChR imaging for peripheral nerve injury and needs to be investigated further. Furthermore, a larger comparative study between alpha7-nAChR imaging and conventional modalities, such as electrophysiology, should be conducted before applying ^18^F-ASEM PET/MRI to patients with peripheral nervous system disorders.

## Conclusions

In conclusion, ^18^F-ASEM PET/MRI revealed significantly increased alpha7-nAChR expression in denervated muscles relative to control muscles in a mouse model of sciatic nerve injury. Thus, ^18^F-ASEM PET/MRI has potential as a noninvasive imaging modality for the evaluation of muscle denervation after nerve injury.

### Supplementary Information

**Additional file 1.** Experimental design.

## Data Availability

The datasets used and/or analysed during the current study are available from the corresponding author on reasonable request.

## Abbreviations

nEMG: Needle electromyography
MRI: Magnetic resonance imaging
FDG: Fluorodeoxyglucose
PET: Positron emission tomography
nAChR: Nicotinic acetylcholine receptor
VOI: Volume of interest
SUV: Standardized uptake value
ROI: Region of interest
MLA: Methyllycaconitine

## References

[1] Simon NG, Spinner RJ, Kline DG, Kliot M (2016). Advances in the neurological and neurosurgical management of peripheral nerve trauma. J Neurol Neurosurg Psychiatry.

[2] Mary P, Servais L, Vialle R (2018). Neuromuscular diseases: diagnosis and management. Orthop Traumatol Surg Res.

[3] Zochodne DW (2007). Diabetes mellitus and the peripheral nervous system: manifestations and mechanisms. Muscle Nerve.

[4] McDonald CM (2002). Physical activity, health impairments, and disability in neuromuscular disease. Am J Phys Med Rehabil.

[5] Dumitru D, Amato A, Zwarts M (2002). Electrodiagnostic Medicine.

[6] Daube JR, Rubin DI (2009). Needle electromyography. Muscle Nerve.

[7] Moon YE, Kim SH, Choi WH (2013). Comparison of the effects of vapocoolant spray and topical anesthetic cream on pain during needle electromyography in the medial gastrocnemius. Arch Phys Med Rehabil.

[8] Wessig C, Koltzenburg M, Reiners K, Solymosi L, Bendszus M (2004). Muscle magnetic resonance imaging of denervation and reinnervation: correlation with electrophysiology and histology. Exp Neurol.

[9] Kamath S, Venkatanarasimha N, Walsh MA, Hughes PM (2008). MRI appearance of muscle denervation. Skeletal Radiol.

[10] Lee SH, Seo HG, Oh BM, Choi H, Cheon GJ, Lee SU (2019). ^18^F-FDG positron emission tomography as a novel diagnostic tool for peripheral nerve injury. J Neurosci Methods.

[11] Lee SH, Seo HG, Oh BM, Choi H, Cheon GJ, Lee SU (2016). Increased (18)F-FDG uptake in the trapezius muscle in patients with spinal accessory neuropathy. J Neurol Sci.

[12] Lee SH, Oh BM, Lee G, Choi H, Cheon GJ, Lee SU (2014). Feasibility of 18F-FDG PET as a noninvasive diagnostic tool of muscle denervation: a preliminary study. J Nucl Med.

[13] Miledi R (1960). The acetylcholine sensitivity of frog muscle fibres after complete or partial devervation. J Physiol.

[14] Witzemann V, Brenner HR, Sakmann B (1991). Neural factors regulate AChR subunit mRNAs at rat neuromuscular synapses. J Cell Biol.

[15] Tsuneki H, Salas R, Dani JA (2003). Mouse muscle denervation increases expression of an alpha7 nicotinic receptor with unusual pharmacology. J Physiol.

[16] Khan MA, Sahani N, Neville KA, Nagashima M, Lee S, Sasakawa T (2014). Nonsurgically induced disuse muscle atrophy and neuromuscular dysfunction upregulates alpha7 acetylcholine receptors. Can J Physiol Pharmacol.

[17] Horti AG, Kuwabara H, Holt DP, Dannals RF, Wong DF (2013). Recent PET radioligands with optimal brain kinetics for imaging nicotinic acetylcholine receptors. J Labelled Comp Radiopharm.

[18] Chalon S, Vercouillie J, Guilloteau D, Suzenet F, Routier S (2015). PET tracers for imaging brain α7 nicotinic receptors: an update. Chem Commun (Camb).

[19] Horti AG (2015). Development of [(18)F]ASEM, a specific radiotracer for quantification of the α7-nAChR with positron-emission tomography. Biochem Pharmacol.

[20] Gao Y, Kellar KJ, Yasuda RP, Tran T, Xiao Y, Dannals RF (2013). Derivatives of dibenzothiophene for positron emission tomography imaging of α7-nicotinic acetylcholine receptors. J Med Chem.

[21] Lee SJ, Oh SJ, Moon WY, Choi MS, Kim JS, Chi DY (2011). New automated synthesis of [18F]FP-CIT with base amount control affording high and stable radiochemical yield: a 1.5-year production report. Nucl Med Biol.

[22] Markou A, Paterson NE (2001). The nicotinic antagonist methyllycaconitine has differential effects on nicotine self-administration and nicotine withdrawal in the rat. Nicotine Tob Res.

[23] Chilton M, Mastropaolo J, Rosse RB, Bellack AS, Deutsch SI (2004). Behavioral consequences of methyllycaconitine in mice: a model of alpha7 nicotinic acetylcholine receptor deficiency. Life Sci.

[24] Sedgwick P (2015). Confidence intervals, P values, and statistical significance. BMJ.

[25] Hillmer AT, Li S, Zheng MQ, Scheunemann M, Lin SF, Nabulsi N (2017). PET imaging of α_7_ nicotinic acetylcholine receptors: a comparative study of [^18^F]ASEM and [^18^F]DBT-10 in nonhuman primates, and further evaluation of [^18^F]ASEM in humans. Eur J Nucl Med Mol Imaging.

[26] Horti AG, Gao Y, Kuwabara H, Wang Y, Abazyan S, Yasuda RP (2014). 18F-ASEM, a radiolabeled antagonist for imaging the α7-nicotinic acetylcholine receptor with PET. J Nucl Med.

[27] Yang T, Wang D, Chen X, Liang Y, Guo F, Wu C (2021). ^18^F-ASEM imaging for evaluating atherosclerotic plaques linked to α7-nicotinic acetylcholine receptor. Front Bioeng Biotechnol.

[28] Coughlin JM, Rubin LH, Du Y, Rowe SP, Crawford JL, Rosenthal HB (2020). High availability of the α7-nicotinic acetylcholine receptor in brains of individuals with mild cognitive impairment: a pilot study using ^18^F-ASEM PET. J Nucl Med.

[29] Coughlin J, Du Y, Crawford JL, Rubin LH, Behnam Azad B, Lesniak WG (2019). The availability of the α7 nicotinic acetylcholine receptor in recent-onset psychosis: a study using ^18^F-ASEM PET. J Nucl Med.

[30] Wong DF, Kuwabara H, Horti AG, Rubin LH, Behnam Azad B, Lesniak WG (2018). Brain PET imaging of α7-nAChR with [18F]ASEM: reproducibility, occupancy, receptor density, and changes in schizophrenia. Int J Neuropsychopharmacol.

[31] Boswijk E, Bauwens M, Mottaghy FM, Wildberger JE, Bucerius J (2017). Potential of α7 nicotinic acetylcholine receptor PET imaging in atherosclerosis. Methods.

[32] Wang D, Yao Y, Wang S, Zhang H, He ZX (2021). The availability of the α7-nicotinic acetylcholine receptor in early identification of vulnerable atherosclerotic plaques: a study using a novel ^18^F-label radioligand PET. Front Bioeng Biotechnol.

[33] Fan YY, Zhang ST, Yu LS, Ye GH, Lin KZ, Wu SZ (2014). The time-dependent expression of α7nAChR during skeletal muscle wound healing in rats. Int J Legal Med.

