# Supplementary figures and images for: 18 F-ASEM PET/MRI targeting alpha7-nicotinic acetylcholine receptor can reveal skeletal muscle denervation
Source: EJNMMI Res. 2024 Jan 22;14:8. doi: 10.1186/s13550-024-01067-9 (PMC10803689; doi:10.1186/s13550-024-01067-9)

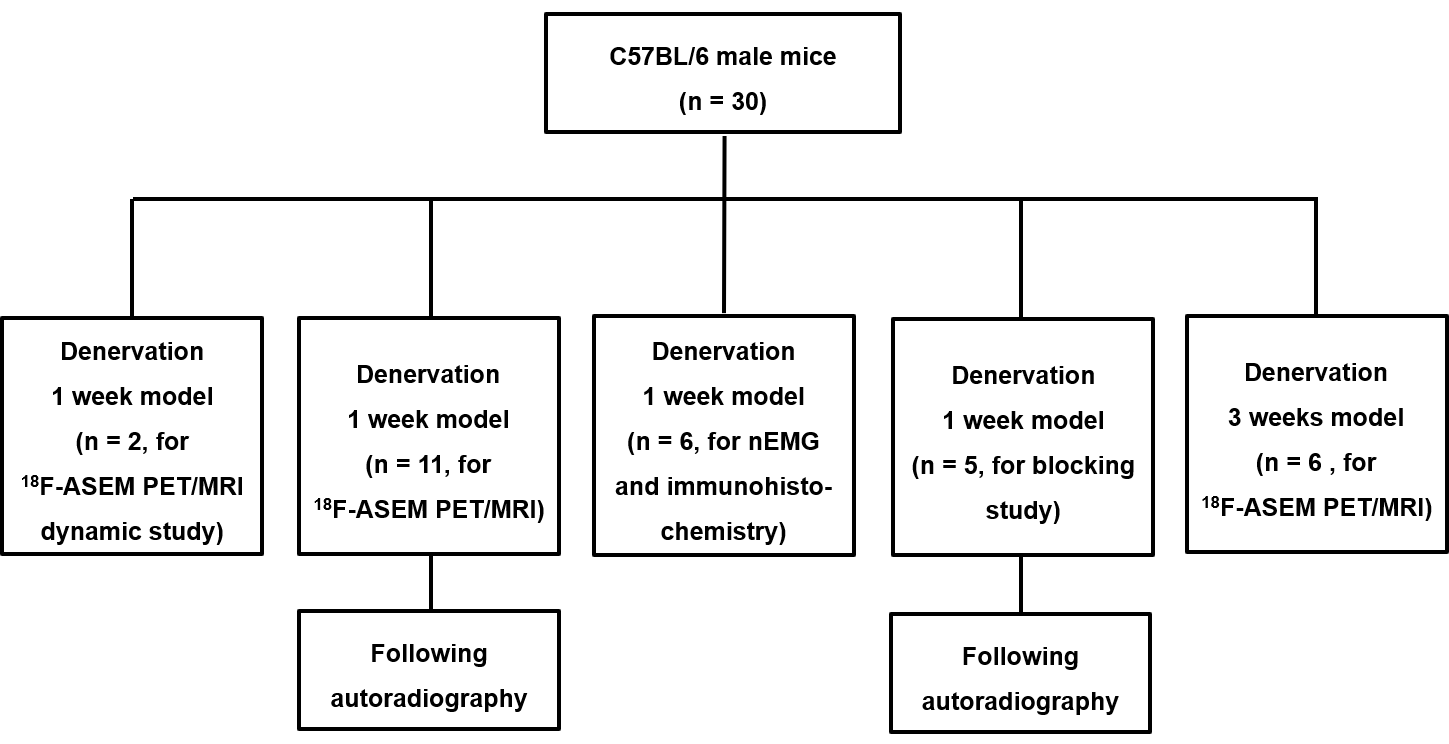

Supplement: Supplementary file 1 — Additional file 1. Experimental design. [file 13550_2024_1067_MOESM1_ESM.tif]
